# Detection of Parent-of-Origin Specific Expression Quantitative Trait Loci by *Cis*-Association Analysis of Gene Expression in Trios

**DOI:** 10.1371/journal.pone.0041695

**Published:** 2012-08-17

**Authors:** Paras Garg, Christelle Borel, Andrew J. Sharp

**Affiliations:** Department of Genetics and Genomic Sciences, Mount Sinai School of Medicine, New York, New York, United States of America; Florida State University, United States of America

## Abstract

Parent-of-origin (PofO) effects, such as imprinting are a phenomenon in which homologous chromosomes exhibit differential gene expression and epigenetic modifications according to their parental origin. Such non-Mendelian inheritance patterns are generally ignored by conventional association studies, as these tests consider the maternal and paternal alleles as equivalent. To identify regulatory regions that show PofO effects on gene expression (imprinted expression Quantitative Trait Loci, ieQTLs), here we have developed a novel method in which we associate SNP genotypes of defined parental origin with gene expression levels. We applied this method to study 59 HapMap phase II parent-offspring trios. By analyzing mother/father/child trios, rules of Mendelian inheritance allowed the parental origin to be defined for ∼95% of SNPs in each child. We used 680,475 informative SNPs and corresponding expression data for 92,167 probe sets from Affymetrix GeneChip Human Exon 1.0 ST arrays and performed four independent *cis*-association analyses with the expression level of RefSeq genes within 1 Mb using PLINK. Independent analyses of maternal and paternal genotypes identified two significant *cis*-ieQTLs (p<10^−7^) at which expression of genes *SFT2D2* and *SRRT* associated exclusively with maternally inherited SNPs rs3753292 and rs6945374, respectively. 28 additional suggestive *cis*-associations with only maternal or paternal SNPs were found at a lower stringency threshold of p<10^−6^, including associations with two known imprinted genes *PEG10* and *TRAPPC9*, demonstrating the efficacy of our method. Furthermore, comparison of our method that utilizes independent analyses of maternal and paternal genotypes with the Likelihood Ratio Test (LRT) showed it to be more effective for detecting imprinting effects than the LRT. Our method represents a novel approach that can identify imprinted regulatory elements that control gene expression, suggesting novel PofO effects in the human genome.

## Introduction

Genomic imprinting is an epigenetic mechanism that modifies the expression of genes dependent on their parental origin. To date ∼60 imprinted genes have been identified in human (http://www.geneimprint.com/), while some studies in mice have suggested that as many as 3% of genes show evidence of imprinting [1]. Many imprinted genes are known to play important roles in growth and development, and disruption of normal imprinting leads to a variety of genetic syndromes such as transient neonatal diabetes [2], Beckwith-Wiedemann syndrome [3], and Prader-Willi syndrome [4]. However, there is a considerable body of evidence indicating that imprinting effects also contribute towards the risk of many common diseases, with nearly 100 entries listed in the Catalog of Parent of Origin Effects [5]. Potential parent-of-origin (PofO) effects have been reported in many other disorders, including juvenile idiopathic arthritis [6], asthma [7], bipolar disorder [8], [9], pre-eclampsia [10], and cleft lip [11], [12].

Recent studies have yielded robust evidence that imprinting effects may be pervasive in common disease. Most prominently, a systematic re-evaluation of association studies in three diseases performed in >38,000 Icelanders identified a number of alleles within known imprinted loci that showed significant PofO effects influencing the risk of breast cancer, basal-cell carcinoma and type 2 diabetes [13]. A similar analysis of >7,500 type 1 diabetes cases also identified a risk allele within the imprinted region on chromosome 14q32.2 that showed reduced paternal transmission, suggesting a parent or origin influence on disease risk [14]. And recently an expression quantitative trait locus (eQTL) of the maternally expressed transcription factor *KLF14* was found to act as a master *trans*-regulator of adipose gene expression, influencing multiple metabolic phenotypes [15].

Array based eQTL studies in HapMap population have identified many regulatory variants that show associations with gene expression levels. By performing an association study using expression data from lymphoblastoid cell lines (LCLs) from four HapMap populations, Stranger et. al (2007) found 1,348 probe sets that had strong association signals with *cis*-linked SNP and copy number variants [16], [17]. Consistent with these results, more recent studies using RNA-seq expression data that provides improved resolution of expression identified hundreds of sequence variants that correlated with variation in mRNA splicing, gene expression and methylation levels [18]–[20]. Many of these variants correspond to the same SNPs identified by genome-wide association studies (GWAS) that act as risk factors for common diseases, demonstrating a functional link between SNPs, gene expression variation and human phenotypes [21]. Although many previous studies have sought to identify eQTLs that modify gene expression levels, conventional association study approaches consider the two parental alleles to be functionally equivalent. In the case of imprinted genes that are expressed differentially from the maternal and paternal alleles this assumption is clearly invalid. Therefore in order to identify potential PofO effects on gene expression an analysis that considers the parental origin of each allele is required.

Previously, the family-based Transmission Disequilibrium Test (TDT) has been used to identify PofO effects influencing complex diseases such as cleft lip/palate [22] and polycystic ovary syndrome [23]. Here, biased transmission of maternal versus paternal alleles to affected offspring suggests a potential PofO effect [24]. Though these tests are robust to population substructure, the TDT ignores between family variations, which contain considerable association information and thus, can result in low power [25], [26]. Kong et al. (2009) implemented a novel analysis approach to analyze the contribution of PofO effects within imprinted regions of the genome to three complex disorders. Using extended pedigree records available for the Icelandic population they were able to define the inheritance of alleles in each individual, and then performed independent associations for maternal and paternal alleles to detect PofO effects on disease risk [13]. However, no prior studies have set out to characterize PofO regulatory effects on gene expression.

In order to address this issue, we describe a novel approach for identifying eQTLs that show a PofO bias. The rationale behind our method is that genes that show a parental bias in expression will undergo differential regulatory effects from the maternal and paternal alleles. Since standard association studies treat both alleles equally, they are severely underpowered to detect effects such as imprinting in which the two alleles are differentially regulated. Here we have developed an association method that is sensitive to parental origin and show that by applying this to analyze 59 HapMap trios for which genome-wide SNP and gene expression data are available we can identify putative imprinted eQTLs (ieQTLs). At its heart our methodology is based on the use of trio data, which enables the definition of parental origin for each allele in the offspring by simple rules of Mendelian inheritance. We show that by utilizing this approach, the separate association of the two parental genotypes with gene expression levels can identify regulatory loci that show putative PofO specific effects that are missed by conventional eQTL analyses, potentially identifying many novel subtle imprinting effects on gene expression across the genome.

## Results

**Figure 1:**
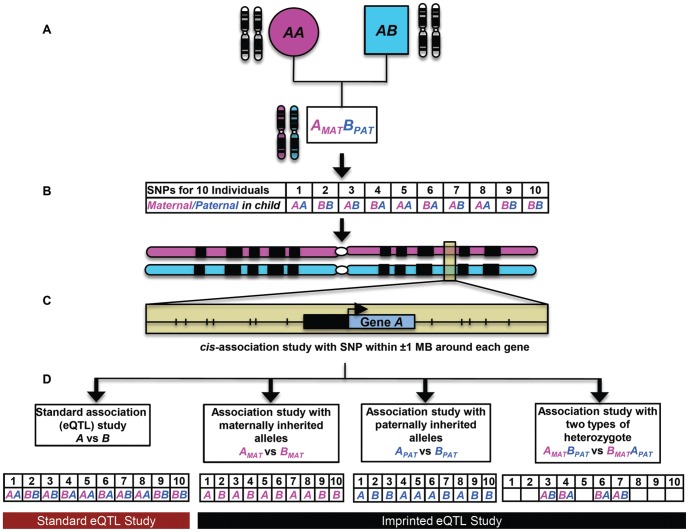
Example of determination of parental origin and parent-of-origin specific association testing for a hypothetical SNP. (A) For the given SNP (alleles *A* and *B*), the homozygous mother can only contribute allele *A* to her offspring, which means that the child's *A* allele is maternally inherited, and child's *B* allele must therefore be paternally inherited. Maternal (_MAT_) and paternal (_PAT_) alleles are shown in pink and blue, respectively. (B) Example data showing annotation of parental origin of this SNP in 10 individuals. (C) *cis*-association study with SNPs within ±1 MB around the gene. (D) Comparison of standard eQTL study and imprinted eQTL study (see Methods for details).

Using HapMap Phase II data, we selected 680,475 common autosomal SNPs (out of the ∼3.1 million SNPs) from 29 CEU and 30 YRI HapMap Phase II trios that had ≥10% minor allele frequency and for which ≥90% individuals were successfully genotyped. In the offspring of each trio, we performed *cis*-association analyses between these SNPs and gene expression measurements generated using filtered data from 92,167 exon probe sets (∼43% of the total) on the Affymetrix GeneChip Human Exon 1.0 ST array (see Methods). After assigning parental origin to each allele in the child using rules of Mendelian inheritance, three *cis*-association tests were performed: 1) an association study using only maternally inherited SNPs; 2) an association study using only paternally inherited SNPs; 3) a comparison of the two classes of reciprocal heterozygote in which parental origin is reversed (Figure 1). Results of these first three tests were compared against those generated in a conventional eQTL association study using diploid genotypes without consideration of parental origin. We limited our analysis to *cis*-acting effects, testing only those filtered SNPs that were within a 2 Mb window centered on the midpoint of each exon probe set.

The key test for identifying a PofO effect is the comparison of association results using only maternal and paternal alleles. For imprinted loci that are expressed from only either the maternal or paternal allele, we expect to observe a significant association with gene expression levels for only one of the two parental alleles. For each exon probe set, we kept only the most significant SNP associated with the expression of that probe set i.e. the SNP with best p-value in the association study. For our analysis, we define SNPs that show an association with gene expression at p<10^−7^ as significant associations, while those with p-value between 10^−7^ and 10^−6^ as suggestive associations. At these thresholds, we identified 30 putative *cis*-ieQTLs, with 2 SNPs showing significant associations with probe sets of the genes *SRRT* and *SFT2D2*, and 28 loci with suggestive associations (Table S1). Of these 30 putative *cis*-ieQTLs, 19 loci showed evidence of association with maternally inherited alleles (p<10^−6^, Bonferroni-corrected p<0.001) but no association with paternally inherited alleles (p>0.05, Bonferroni p = 1). Similarly, 11 SNPs associated strongly with the paternally inherited alleles (p<10^−6^, Bonferroni p<0.001) but showed no association with the corresponding maternally inherited alleles (p>0.05, Bonferroni p = 1).

**Figure 2:**
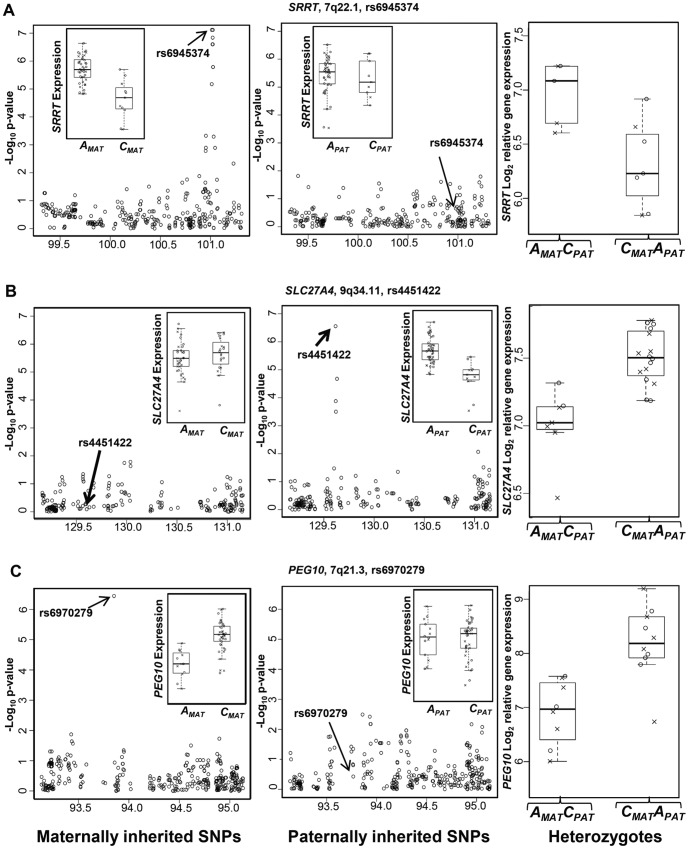
Putative *cis*-ieQTLs detected by parent of origin specific association analysis in 59 HapMap trios. Results for (A) *SRRT* and (B) *SLC27A4* and (C) *PEG10*. In each plot, the panels show the distribution of p-values for each SNP within a ±1 Mb region around each gene based on separate association of the maternally- and paternally-inherited SNPs (left and middle panels respectively). The x-axis shows the genomic coordinates in Mb, and y-axis is the –log_10_ p-value of association between each SNP allele of defined parental origin and gene expression level. Box plots show the distributions of gene expression for CEU (*circles*) and YRI offspring (*crosses*) with respect to maternally (_MAT_) and paternally (_PAT_) inherited SNPs. A clear difference between heterozygous individuals of reciprocal parental origin is also consistent with a PofO effect at these loci, as these individuals show a difference in gene expression despite having the same genotype (right panels).

The two most prominent PofO signals we identified were that of a maternal-specific association between alleles of rs6945374 and the expression level of *SRRT* (Maternal-specific p = 7.6×10^−8^, Bonferroni p = 2.5×10^−5^; Paternal specific p = 0.59, Bonferroni p = 1, Figure 2A), and an association between maternally-derived alleles of rs3753292 and *SFT2D2* expression (Maternal-specific p = 2.1×10^−8^, Bonferroni p = 9.6×10^−6^; Paternal-specific p = 0.37, Bonferroni p = 1). The strongest paternal-specific association identified was between alleles of rs4451422 and *SLC27A4* expression level (Paternal-specific p = 2.8×10^−7^, Bonferroni p = 7.4×10^−5^, Figure 2B), while maternally derived alleles of this SNP were not significant (p = 0.42, Bonferroni p = 1).

Of the 30 *cis*-ieQTLs that showed suggestive PofO specific signals, two were in association with the known imprinted genes *PEG10* and *TRAPPC9*. SNP rs6970279, which lies 270 kb upstream of *PEG10* showed a suggestive association specifically with the maternally-derived allele (p = 3.6×10^−7^, Bonferroni p = 1.3×10^−4^). In contrast, the paternally-derived genotype has no significant influence on *PEG10* expression (p = 0.87, Bonferroni p = 1, Figure 2C). Similarly, expression levels of the imprinted gene *TRAPPC9* [27] were strongly correlated with the SNP rs7003899 when inherited paternally (p = 8.9×10^−7^, Bonferroni p = 0.0004), while there was no such correlation with the same SNP when inherited maternally (p = 0.64, Bonferroni p = 1). Based on the number of known imprinted genes in human and the probe sets utilized in our analysis, the identification of two known imprinted genes in the 30 *cis*-ieQTLs we describe represents a 24.4-fold enrichment compared to that expected under the null hypothesis (p = 7.7×10^−5^).

In addition to association testing using only the maternally- and paternally-derived alleles, we also performed a test comparing expression levels between the two reciprocal classes of heterozygote (*A_MAT_B_PAT_* versus *A_PAT_B_MAT_*, Figure 1). Since reciprocal heterozygotes have identical genotype but reversed parental origin, any expression bias between these two groups is strongly indicative of an underlying PofO effect. Although with large sample sizes this represents a robust method to assay for PofO effects, the small size of our study population (n = 59 offspring) resulted in limited numbers of loci at which sufficient numbers of both heterozygote classes were available. We therefore did not utilize this test as a primary screening tool, but rather as secondary evidence in support of our association testing using maternal and paternal alleles. At a threshold of p<10^−7^, there were no significant associations using this heterozygote test. However, we identified 7 suggestive associations that showed a difference in gene expression between the reciprocal heterozygote classes at p<10^−6^ (Table S2). Of the 30 *cis*-ieQTLs that were identified by association with only maternal or paternal alleles, 24 of these yielded moderate p-values with the heterozygote test (p-values ranging from 1.1×10^−5^ to 3.5×10^−2^). For the remaining 6 *cis*-ieQTLs there were insufficient numbers of heterozygous individuals (n<8) to perform the test.

We compared the results of above three PofO specific association studies with those gained in a conventional *cis*-eQTL study that does not consider the parental origin of alleles. Of the 30 putative *cis*-ieQTL loci we report, only 2 achieved suggestive significance in a standard *cis*-eQTL test (p<10^−6^) (Table S1), showing that our method detects novel loci that are not detected when parental origin is not considered.

To compare our methods with a previously published approach for detecting PofO effects, we also analyzed our genome-wide dataset using the Likelihood Ratio Test (LRT), a test which compares an imprinting model vs. a non-imprinting model (see Methods). Using the LRT, the coefficients of linear regression represent the contribution of a variable (in this case SNP alleles) to variation in gene expression. For the 19 maternal ieQTLs identified in our analysis, the absolute ß_M_ using the LRT ranged from 0.282 to 1.17 while absolute ß_P_ ranged from 0.001 to 0.292. This indicates that the variation in gene expression is better explained by maternally inherited alleles compared to paternally inherited alleles at these loci, consistent with a PofO effect. Similarly, for paternal ieQTLs, the absolute ß_P_ ranged from 0.321 to 1.089 compared to ß_M_, which ranged from 0.011 to 0.157. In each case, the coefficient of linear regression without considering imprinting (ß′_B_) was intermediate between ß_M_ and ß_P_, consistent with the idea that the independent effects of maternal and paternal alleles on gene expression are underrepresented in the reduced model (Table S1).

**Figure 3:**
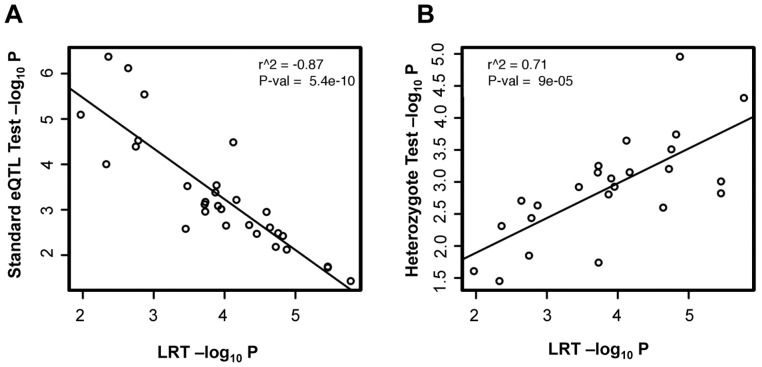
Comparison of separate association using maternal and paternal alleles with the Likelihood Ratio Test (LRT) for putative 30 ieQTLs. (A) Correlation between –log_10_ p-values generated using a standard association test and the LRT (r^2^ = −0.87) (B) Correlation between -log_10_ p-values generated using the heterozygote test and the LRT (r^2^ = 0.71). Note that 6 of the 30 SNP-gene pairs did not have a sufficient number of heterozygotes to perform the test.

For the 30 ieQTLs identified using our association test with maternal and paternal alleles, we found that the (-log_10_) p-values obtained from the LRT for each respective SNP-gene pair showed a strong negative correlation (r^2^ = −0.82) with the (-log_10_) p-values obtained using a conventional association test that does not consider parental origin. Conversely, results of the LRT showed a strong positive correlation with the (-log_10_) p-values generated in the heterozygote test (Figure 3, Table S2). For maternal-specific associations in the 30 ieQTLs, results of the LRT showed that the (absolute) coefficient of regression for maternal alleles was greater than for paternal alleles and vise-versa for paternal specific associations. However, although all 30 ieQTLs identified by our maternal and paternal specific association test showed at least nominal significance (p<0.05) with the LRT, many were non-significant using the LRT after multiple testing correction. Furthermore, unlike the independent analyses of maternal and paternal genotypes, results of a genome-wide screen with the LRT did not detect any associations at p<10^−6^ with known imprinted genes (Table S3).

## Discussion

We have developed a novel method that allows the genome-wide identification of ieQTLs that show regulatory effects on gene expression in a PofO specific manner. By using SNP genotype and gene expression datasets in HapMap trios, we show that the independent association of maternal and paternal alleles with offspring phenotypes can identify functional genetic variants that are invisible to standard association studies that do not consider parental origin. Implementation of our method reveals two putative maternal-specific *cis*-ieQTLs that are significantly associated with the expression level of *SRRT* and *SFT2D2* (p<1×10^−7^), and an additional 28 loci that yielded suggestive associations (p-values between 10^−6^ and 10^−7^). In each case, these SNPs showed a strong association with just one parental allele, and no association (p>0.05) with other parental allele. These associations suggest possible *cis*-PofO effects of these loci on nearby gene expression. Furthermore, only 2 of these 30 ieQTLs were detected (at p<10^−6^) using a conventional eQTL association study that does not consider parental origin, showing that our method identifies novel loci that fail to reach statistical significance using tests that ignore parental origin. As standard association studies make no distinction between maternal and paternal inheritance, they have the inherent presumption that both alleles of the diploid genome are functionally equivalent. By combining the effects of both maternal and paternal alleles together equally, this can therefore mask differential effects that may be present between the two parental alleles. Thus, most imprinting effects are masked using standard association study approaches, demonstrating that specialized methods that consider the parental origin, such as we describe here, are necessary to identify such regulatory elements with PofO effects.

We also performed a third association test based on relative gene expression in heterozygous individuals with identical genotypes but which are of opposite parental origin (*A_MAT_B_PAT_* versus *A_PAT_B_MAT_* in Figure 1). A similar idea has been used to search for PofO differences in allelic expression based on the study of expression bias in individuals heterozygous for coding SNPs [28]. The main advantage of this heterozygote test lies in the fact that the two types of heterozygote have identical genotypes but their parental origin is reversed. Thus, any difference detected between these two groups strongly suggests an underlying PofO or epigenetic effect on gene expression. Our implementation of this heterozygote test in HapMap CEU and YRI trios identified 7 associations with a suggestive difference in gene expression (p<10^−6^). However, the utility of this test in our analysis was severely limited as there was an average of only 10 heterozygous individuals per filtered SNP, resulting in low statistical power. As a result, we chose not use results of the heterozygote test as a primary screen for ieQTLs, but instead as supporting evidence for loci detected by the comparison of the two parental alleles.

The main limitation in this study was the relatively small number of HapMap trios (n = 59) for which both genome-wide SNP and gene expression data were available. In every GWAS, the power to detect significant associations is strongly influenced by the number of individuals studied [29], and the sample size utilized here probably represents the absolute minimum required in a study of this type, giving sufficient power to detect only variants of relatively large effect. Furthermore, our small sample size can also result in a high false positive rate. Despite these caveats, of the 30 putative ieQTLs we report, our method was successfully able to identify two associated with the known imprinted genes *PEG10* and *TRAPPC9*. Given the relatively small number of known imprinted genes known in human, this represents a 24.4-fold enrichment for imprinted genes in our ieQTL set above that which would be expected by random chance (p = 7.7×10^−5^), strongly suggesting that our methodology is able to successfully identify regulatory elements that function in a PofO specific manner.

**Figure 4:**
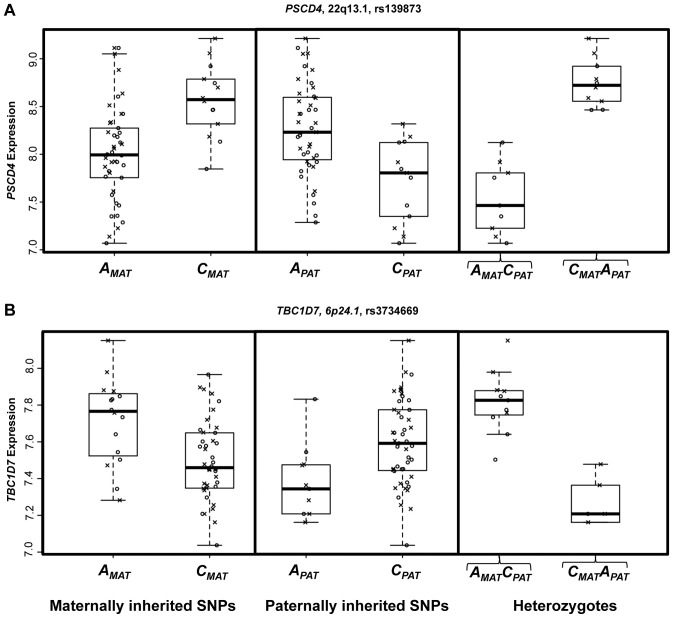
The two most significant SNP-gene pairs identified using the Likelihood Ratio Test. Results for (A) *PSCD4* and (B) *TBC1D7*. In each plot the three panels show variation of gene expression with respect to maternally inherited SNPs, paternally inherited SNPs and two classes of heterozygotes, respectively. Box plots show the distributions of gene expression for CEU (*circles*) and YRI offspring (*crosses*) with respect to maternally (_MAT_) and paternally (_PAT_) inherited SNPs. Note that unlike loci identified using separate association with maternal and paternal alleles where a difference in gene expression level is seen for only one of the two parental alleles, for both genes the A and C alleles have opposite effects on gene expression when inherited maternally versus paternally.

We compared results of our method that looks for associations specific to one parental allele with those identified using the Likelihood Ratio Test. This method to compare an imprinting model against a non-imprinting model has previously been suggested as robust for detecting PofO effects [30]. In support of this, all 30 putative ieQTLs identified by our PofO method showed at least nominal significance (p<0.05) with the LRT, and there was a strong positive correlation between results of the heterozygote test and the LRT. However, although both methods identified similar numbers of putative PofO-specific associations, there was no overlap between the most significant loci identified by our novel methods and those obtained by the LRT. Inspection of the data at loci identified by these alternate methods for identifying PofO effects revealed a strong difference between them in the relative distributions of gene expression levels associated with the two parental alleles. As our novel method looks for associations specific to just one parental allele, the loci we identify such as the SNP rs6970279 associated with the imprinted gene *PEG10* show a strong difference in gene expression for alleles inherited from one parent, while alleles from the other parent show no effect on expression levels (Figure 2). In contrast, at loci scored the most highly significant using the LRT, alleles inherited from each parent tend to show reciprocal effects on gene expression levels to one another (Figure 4). Given that imprinted regulatory elements will generally act on only one of the two parental alleles, rather than showing opposite effects when inherited maternally versus paternally, we suggest that our method is therefore better suited for identifying genuine ieQTLS compared to the LRT. This is supported by the fact that unlike our method which identified ieQTLs associated with both *PEG10* and *TRAPPC9*, application of the LRT genome-wide failed to identify any known imprinted genes.

In summary, we have developed a novel approach in which separate association studies for the two parental alleles allow genome wide detection of regions that influence gene expression in a PofO specific manner. Our data suggests that each of these loci contains a regulatory element that influences gene expression on only one of the two parental alleles, such as an imprinted locus. As conventional association analyses presume both homologous chromosomes to be functionally equivalent, these sites are generally undetected by standard association study approaches. The comparison of reciprocal classes of heterozygotes represents an additional test that further supports the PofO effects of those regions, though this test requires adequate sample size to have sufficient power. Using published datasets from HapMap trios, we were able to identify putative differential effects of the maternally and paternally inherited alleles on gene expression. Since many PofO effects and eQTLs are tissue specific, we cannot generalize our results for all cell types. For example, while *SNRPN* is widely expressed in most tissues, in contrast *NDN* is silent in LCLs [31], [32], [33]. We anticipate that future applications of this methodology, perhaps including different tissue types such as placenta in which imprinting effects are frequent, will allow the identification of many additional loci in the human genome that exhibit PofO bias. Furthermore, we suggest that our methods could also be modified to detect PofO effects in a variety of common diseases.

## Materials and Methods

### Genotype data

We downloaded genotypes for 3,849,034 and 3,881,498 non-redundant autosomal SNPs of 59 trios (mother/father/child) for CEU (CEPH Utah) and YRI (Yorubans), respectively from HapMap Phase II (release 24, http://hapmap.ncbi.nlm.nih.gov) [34]. In each population, SNPs with <10% Minor Allele Frequency (MAF) and <90% genotyped individuals were removed from further analysis. Additionally, as we combined data from both CEU and YRI in our final analysis, SNPs whose MAF differed significantly between the two populations (p<0.05, Fisher's exact test) were removed to avoid effects resulting from population stratification. After filtering, 680,475 SNPs from 59 trios remained and were used for subsequent association analysis (Figure S1).

### Gene expression data

Gene expression data for 29 CEU and 30 YRI children generated by Zhang et al. using Affymetrix GeneChip Human Exon 1.0 ST arrays [35] that was pre-processed by first removing probes that overlapped known SNPs, background correction, quantile normalization and log_2_ transformation, were downloaded from Gene Expression Omnibus (www.ncbi.nlm.nih.gov/geo/, GSE9703). Array data was summarized at the exon level before filtering to remove low expressed and invariant exons (intensity log_2_<6, standard deviation <0.2). To avoid artifacts resulting from population stratification of gene expression, in subsequent association analysis we excluded any exon that had significantly different expression between the CEU and YRI populations (p<0.05, unpaired student's t-test). After these filters, probes from 97,724 exons remained. Using mapping information provided by Affymetrix [GEO accession: GPL5188], we intersected these exons with RefSeq genes on autosomal chromosomes in hg18 (http://genome.ucsc.edu/), keeping only those with at least 1 bp overlap. The final dataset comprised 92,167 exons in the 59 CEU and YRI offspring that were used for association analysis (Figure S1).

### Assigning parental origin to SNPs in each offspring

The rule of Mendelian inheritance allows assignment of parental origin to most SNPs in a child by considering a mother/father/child trio and applying simple logic tests. In the absence of phased genotypes, at least one homozygous genotype at a particular SNP in the trio is required to assign parental origin in the child while rest were removed from the analysis. At each site, a homozygous member of the trio was used to define the parental origin in child for that SNP (see Figure 1A, 1B for details). Although phased genotypes are available for the HapMap, we chose to use unphased data to demonstrate the utility of our methodology in the absence of phase information. The use of phased data would result in a slight increase in overall power by allowing parental origin to be defined for those sites at which all three members of a trio are heterozygous (representing ∼5% of genotypes per trio).

### Cis-association studies

For each SNP, three association tests with gene expression levels in *cis* were performed using data from the 59 CEU and YRI offspring: 1) an association study using only the maternally inherited alleles; 2) an association study using only the paternally inherited alleles [13] 3) a comparison of expression between the two classes of heterozygote with reciprocal parental origin (*A_MAT_B_PAT_* versus *A_PAT_B_MAT_* in Figure 1). As a comparison, a conventional association test was also performed with diploid genotypes without using parental origin information. Custom Perl scripts were used to convert the data into PLINK (v1.07) format (MAP and PED files). Since PLINK requires input SNPs to be in a diploid format, for the maternal and paternal allele tests in which haploid genotypes are utilized, we converted these haploid genotypes into an artificial homozygous diploid state that would be accepted by PLINK. Similarly, as the two classes of heterozygote with opposite parental origin have identical genotypes, we converted these genotypes to opposing diploid homozygous states for input to PLINK to perform the heterozygote test. Mendelian error (–me) and Hardy-Weinberg Equilibrium (–hwe) exclusion thresholds were set to zero for these tests to ensure that these artificial diploid genotypes would pass these quality filters. For association testing, SNPs within a ±1 Mb window from each exon mid-point were used [36] and nominal p-values for each SNP-gene pair in the four statistical tests were recorded (–assoc option in PLINK) (Figure 1C, 1D). A Bonferroni correction was applied to account for the fact that multiple SNPs were associated with each probe set.

### Identification of Imprinted eQTLs

We defined an ieQTL as a SNP that shows association with either the maternally- or paternally-derived alleles. To control for family-wide type I error, we considered associations with p<10^−7^ as “significant" while those with p-value between 10^−6^ and 10^−7^ as “suggestive" [37]. As we were interested only in sites that showed PofO effects, we required each putative ieQTL to have p>0.05 (i.e. no evidence of association) with the other parental allele. Because of the LD structure of the genome, many loci showed significant associations with multiple closely spaced SNPs. To avoid such redundancies, we only reported the most significant association for each probe set with nominal and corrected p-values in Tables S1, S2, S3.

### Testing an imprinting model using the Likelihood Ratio Test

We designed a likelihood ratio model to identify parent-of-origin effects in gene expression, in which we performed linear regression using maternally and paternally inherited alleles as independent variables. This model was tested against the null model of both alleles combined (i.e. ignoring parental origin of alleles). This test results in more significant p values when both maternal and paternal alleles have differential effects on the gene expression,
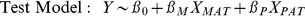
(1)

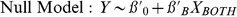
(2)where, Y is the gene expression, X_MAT_ and X_PAT_ are minor alleles inherited from the mother and father respectively and *X_BOTH_* is the count of minor allele in child for a particular SNP i.e. sum of X_MAT_ and X_PAT_. The coefficients of regression *ß_0_* and *ß′_0_* correspond to average expression level. *ß_M_* and *ß_P_* are variation of expression explained by maternal and paternal inheritance respectively while ß′_B_ is the variation explained when no parental origin is considered.

### Enrichment of known imprinted genes in ieQTLs

Coordinates for experimentally verified human imprinted genes were downloaded from GeneImprint (http://www.geneimprint.com/), The Catalogue of Parent of Origin Effects (http://igc.otago.ac.nz/), and from a survey of the literature, to form table of all known imprinted genes. This list of verified imprinted genes was intersected with the filtered Affymetrix exons mapping to known imprinted genes. The significance of enrichment was calculated using a Hypergeometric distribution.

## Supporting Information

Figure S1 Flowchart showing data filtering for SNP genotype and Affymetrix data. After quality filtering for SNPs and probes, 680,475 SNPs and expression data from 92,167 probe sets were utilized from the 59 CEU and YRI trios were used for ieQTL analysis.
(TIF)

Table S1 Results for 30 putative ieQTLs identified by *cis*-association analysis of gene expression levels with maternal and paternal alleles.
(XLS)

Table S2 Results for 7 SNPs identified by comparison of expression levels between reciprocal heterozygotes.
(XLS)

Table S3 Results for 42 *cis*-eQTLs identified using the Likelihood Ratio Test (LRT).
(XLSX)
