# Supplementary figures and images for: Detection of Parent-of-Origin Specific Expression Quantitative Trait Loci by Cis-Association Analysis of Gene Expression in Trios
Source: PLoS One. 2012 Aug 17;7(8):e41695. doi: 10.1371/journal.pone.0041695 (PMC3422236; doi:10.1371/journal.pone.0041695)

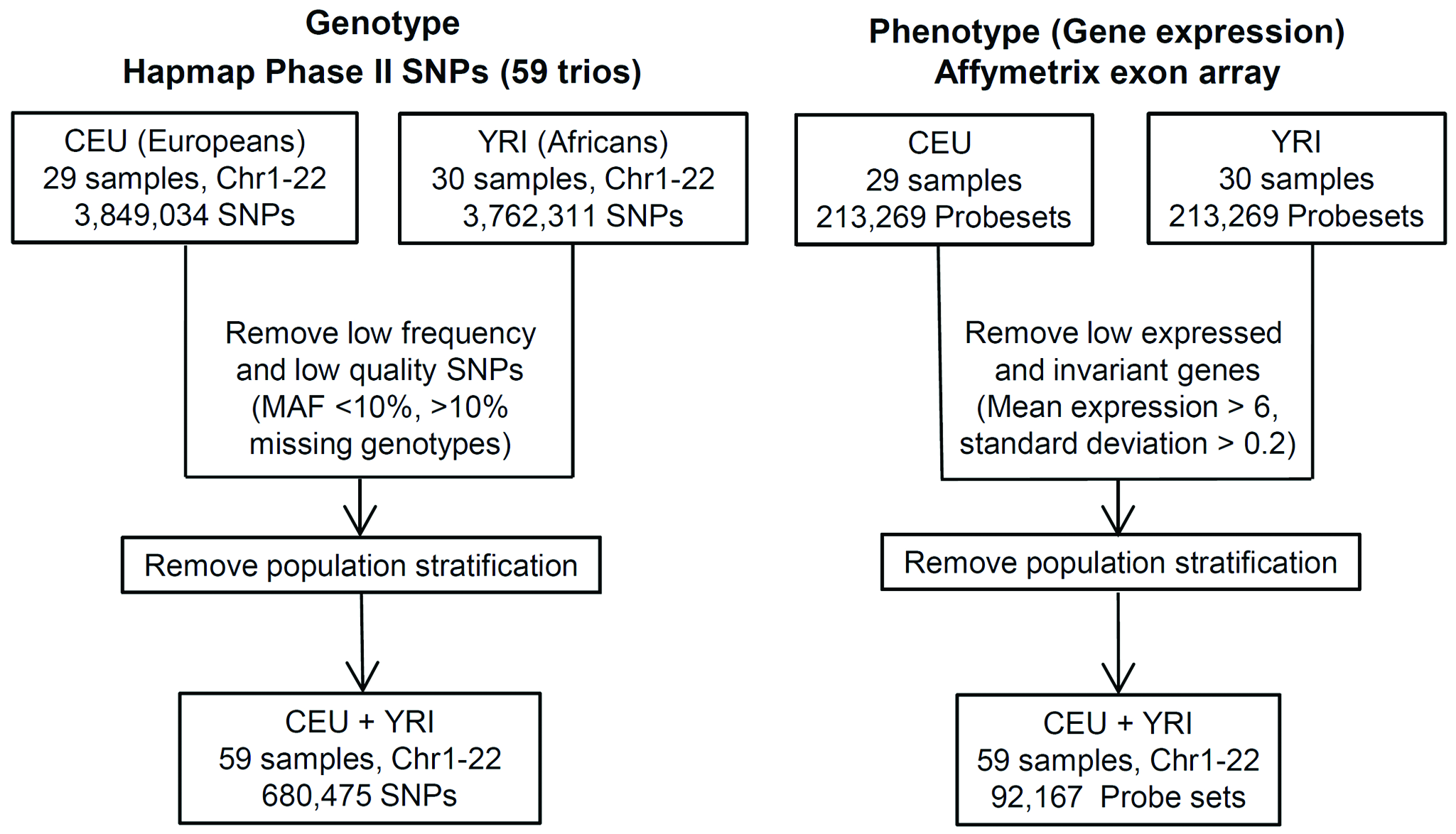

Supplement: Figure S1 — Flowchart showing data filtering for SNP genotype and Affymetrix data. After quality filtering for SNPs and probes, 680,475 SNPs and expression data from 92,167 probe sets were utilized from the 59 CEU and YRI trios were used for ieQTL analysis. (TIF) [file pone.0041695.s001.tif]
